# The use of biomarkers testing in Emergency Department

**DOI:** 10.2478/jccm-2024-0041

**Published:** 2025-04-30

**Authors:** Sonia Luka, Adela Golea, Raluca Mihaela Tat, Eugenia Maria Lupan Mureșan, George Teo Voicescu, Ștefan Cristian Vesa, Maria Adriana Albu, Daniela Ionescu

**Affiliations:** Faculty of Medicine, Iuliu Hagieganu University of Medicine and Pharmacy, Cluj-Napoca, Romania; Clinical Emergency County Hospital, Cluj-Napoca, Romania; Discipline of Emergency Medicine, Faculty of Medicine, Iuliu Hatieganu University of Medicine and Pharmacy, Cluj-Napoca, Romania; CRIMEDIM Center for Research and Training in Disaster Medicine, Humanitarian Aid and Global Health, Università del Piemonte Orientale, Novara, Italy; Discipline of Pharmacology, Toxicology and Clinical Pharmacology, Faculty of Medicine, Iuliu Hatieganu University of Medicine and Pharmacy, Cluj-Napoca, Romania; Department of Emergency Medicine and First Aid, “Carol Davila” University of Medicine and Pharmacy, Bucharest, Romania; Discipline of Anaesthesia and Intensive Care I, Faculty of Medicine, Iuliu Hatieganu University of Medicine and Pharmacy, Cluj-Napoca, Romania

**Keywords:** biomarker, emergency department, point-of-care testing, early detection, emergency management

## Abstract

**Introduction:**

In the fast-paced environment of Emergency Departments (EDs), biomarkers are essential for the rapid diagnosis and management of critical conditions.

**Aim of the study:**

This study evaluates the current clinical practice on key biomarkers in Romanian EDs, addressing the needs of emergency medicine physicians, and the challenges associated with biomarker testing.

**Material and Methods:**

An online survey was sent to physicians working in ED to explore their perceptions, needs, and barriers regarding biomarkers, including Point-of-care (POC). Data was collected anonymously through an online platform and subsequently analyzed.

**Results:**

This survey analyzed data from 168 completed responses, with 95.2% of respondents being specialists in emergency medicine. Procalcitonin and presepsin were most preferred for PoCT, while troponin and D-dimer were highly rated regardless of the testing method, reflecting their utility in sepsis and cardiovascular emergencies. Neuron-specific enolase, interleukin-6, and procalcitonin were the biomarkers considered needed.

**Conclusions:**

The most frequently used biomarkers in ED were troponin, D-dimer, BNP/NT-proBNP, and procalcitonin. NSE, IL-6, and procalcitonin were the most recommended for future integration. High costs, limited availability, and false-positive concerns remain significant challenges in biomarker use.

## Introduction

Biomarker testing has become increasingly vital in Emergency Departments (EDs) since its first use in the late 1990s with the introduction of cardiac biomarkers like troponin for diagnosing acute myocardial infarction [1]. At present, a critical component of emergency diagnostics, biomarker testing is employed to rapidly and accurately diagnose and stratify the severity of acute conditions, such as sepsis and heart failure, facilitating timely clinical decision-making [2].

In the high-pressure patients flow of the ED, where time-sensitive decisions are essential, biomarkers testing may offer rapid diagnostic insights, risk stratification, and treatment guidance [3–4]. On the other hand, point-of-care testing (PoCT) is preferred for its ability to provide immediate results, optimize decision-making, reduce reliance on laboratories, and improve patient flow and resource utilisation in overcrowded Ed [5,6,7]. By contrast, laboratory testing is utilized when greater analytical precision is required or when the urgency is lower, allowing for more comprehensive analysis [8] or when biomarkers are not available as PoCT or not frequently used. This study, included a questionnaire distributed to emergency medicine physicians and other specialties working in EDs, aimed to identify the most used biomarkers in emergency settings.

We focused on traditional biomarkers such as troponin [9], natriuretic peptide tests (BNP, NT-proBNP) [10], d-dimer [11], procalcitonin [12], and C-reactive protein (CRP) [12], as well as promising inflammation and prognostic biomarkers like interleukin-6 (IL-6) [13] and presepsin [14]. These biomarkers have an important role for the rapid identification and management of most frequent critical conditions met in ED such as myocardial infarction, sepsis, and pulmonary embolism.

Study aimed to explore the current practice in ED and the needs for additional biomarkers as well as preferences of physicians working in EDs by assessing the current utilization of PoCT biomarkers, while identifying gaps in their availability.

## Materials and Methods

### Study design

The study was observational, non-invasive, non-intervention and non-interactive. Study was approved by the Ethics Committee of the “Iuliu Hațieganu” University of Medicine and Pharmacy Cluj-Napoca. The questionnaire was also approved by the chairs of the emergency departments involved in its distribution.

An electronic questionnaire was distributed among physicians working in ED, including senior and junior physicians, (4^th^ and 5^th^ year residents). Thus, targeted specialties were emergency medicine, intensive care, general practice, and pediatrics, certified in emergency medicine. The questionnaires were disseminated by email via an online link (https://www.surveymonkey.com/r/2XGFLGK), and data were automatically collected using SurveyMonkey Inc. (San Mateo California, USA, www.surveymonkey.com).

The questionnaire, available in Romanian language, was distributed online between August 28^th^ and September 4^th^ to the heads of EDs across 10 major counties in Romania, targeting teaching and county hospitals from all geographical regions of Romania. Approximately 400 practitioners work in these EDs. We focused our study on EDs because it has the highest patient turnover, evaluates the most patients daily, and relies on biomarker testing for rapid diagnosis and management of critical conditions [15].

Only fully completed questionnaires were included in the statistical analysis. The response rate of 43.5%. The questionnaire included questions addressed to current practice on biomarkers as well as to what assay biomarkers were considered necessary to be introduced into regular practice. Questions on demographic data of the responders were also registered. We used a 5-point Likert scale to assess responses, ranging from 1 to 5, where 1 indicated “very rare,” 2 “rare,” 3 “neither often nor rare,” 4 “often,” and 5 “very often” for five of the questionnaire questions regarding the frequency of biomarker use and the most common diseases they aid in diagnosing.

### Statistical analysis

The statistical analysis of this study was done using MedCalc^®^ Statistical Software version 23.0.2 (MedCalc Software Ltd, Ostend, Belgium; https://www.medcalc.org; 2024). Descriptive statistics, including frequencies, percentages, medians and 25–75 percentiles, were used to summarize the data. The Kruskal-Wallis was used to assess differences between groups. The significance level for all statistical tests was set at p < 0.05.

## Results

Of the 174 questionnaires completed by doctors, 6 provided incomplete answers, leaving 168 responses to be analyzed. Table 1 summarizes the demographics of the respondents: 42.9% were senior and 24.4% junior physicians, primarily in Emergency Medicine (95.2%). Most had 1–5 years (29.8%) or 6–10 years (23.8%) of experience. The majority were female (67.3%), aged 31–40 (39.3%) and 41–50 (30.4%). Of the respondents, 88.1% worked in ED, 83.9% were affiliated with university hospitals, and 97.6% in public healthcare, with 67.3% at county hospitals.

**Table 1.** Demographic data of the responders

**Number of answers = 168**	**n (%)**
Professional category:	
Senior physician	72 (42.9)
Junior physician	41 (24.4)
5th year resident	25 (14.9)
4th year resident	30 (17.9)

Specialty:	
Emergency Medicine	160 (95.2)
Family Medicine	3 (1.8)
Anaesthesia and Intensive Care	0 (0)
Paediatrics	4 (2.4)
Other specialty	1 (0.6)

Professional experience (including residency):	
1–5 years	50 (29.8)
6–10 years	40 (23.8)
11–15 years	27 (16.1)
16–20 years	21 (12.5)
21 years or more	30 (17.9)

Gender	
Male	53 (31.5)
Female	113 (67.3)
Prefer not to disclose	2 (1.2)

Age	
20–30 years	27 (16.1)
31–40 years	66 (39.3)
41–50 years	51 (30.4)
51–60 years	24 (14.3)

Type of emergency service	
Emergency Department	148 (88.1)
Emergency Reception Unit	20 (11.9)

Type of work setting:	
Public system	164 (97.6)
Private system	1 (0.6)
Both	3 (1.8)

Type of hospital	
County	113 (67.3)
Municipal	42 (25)
City	3 (1.8)
Military Hospital	4(2.4)
Other Hospital	6 (3.6)

Is the hospital where you work a university hospital (involved in training residents/students)?	
Yes	141 (83.9)
No	27 (16.1)

Physicians reported frequent daily use of biomarkers, with PoCT being referred to as the primary method when both options were available. Biomarkers were rated as most useful for managing cardiovascular conditions, including myocardial infarction 5 (5–5), pulmonary embolism 5 (4–5), heart failure 5 (4–5), and sepsis and septic shock 5 (4–5) on the Likert scale. Among the biomarkers, troponin was rated the highest by the frequency of its use, with a median score of 5 (5–5), followed by D-dimer, BNP/NT-proBNP, and procalcitonin, each scoring a median of 5 (4–5) (Table 2).

**Table 2.** Current practice

**Number of answers = 168**	**Likert scale**	**Median (IQR)**
How frequently do you use biomarkers in your daily practice?	1 – 5	5 (4 – 5)

How often do you consider it necessary to use biomarkers other than those available in your hospital?	1 – 5	3 (3 – 4)

What is the method for biomarker testing in the department where you work?	1 – 5	
The hospital’s central laboratory		
The department’s own laboratory with Point of Care devices Both	1 – 3	2 (2 – 3)

How frequently do you use point of care biomarkers?	1 – 5	4 (4 – 5)

How often do you use biomarkers for the diagnosis of the following critical conditions?		
Acute myocardial infarction	1 – 5	5 (5 – 5)
Pulmonary embolism	1 – 5	5 (4 – 5)
Aortic dissection	1 – 5	5 (4 – 5)
Sepsis and septic shock	1 – 5	5 (4 – 5)
Acute heart failure	1 – 5	4 (4 – 5)
Cerebral hypoxia	1 – 5	3 (1 – 5)
Traumatic brain injury	1 – 5	3 (1 – 4)
Stroke	1 – 5	3 (1 – 4)

Please rank the following biomarkers according to their utility in daily practice:		
Troponin	1 – 5	5 (5 – 5)
D-dimer	1 – 5	5 (4 – 5)
Brain natriuretic peptide or N-terminal pro b-type natriuretic peptide	1 – 5	5 (4 – 5)
Procalcitonin	1 – 5	5 (4 – 5)
C - reactive protein	1 – 5	4 (4 – 5)
Presepsin	1 – 5	4 (3 – 5)
Interleukin - 6	1 – 5	3 (2 – 4)

Physicians rated rapid diagnosis (86.3%), aid in differential diagnosis (86.3%), and improved quality of care (75.6%) as the top benefits of biomarker use in emergency care, with NSE (46.4%), IL-6 (41.7%), and procalcitonin (34.5%) being the most desired for future implementation in clinical practice (Table 3).

**Table 3.** Advantages and emerging biomarker demands in ED

**Number of answers = 168**	**n (%)**
Biomarkers are useful in:	
Differential diagnosis	145 (86.3)
Rapid diagnosis of a condition	145 (86.3)
Enhancing the quality of medical care	127 (75.6)
Assessing the severity of a condition	108 (64.3)
Prognosis	73 (43.5)
Avoiding the administration of unnecessary treatments	72 (42.9)
Streamlining patient flow	71 (42.3)

What is the method for biomarker testing in the department where you work?	
The department’s own laboratory with Point of Care (POC) devices	81 (48.2)
The hospital’s central laboratory	12 (7.1)
Both	75 (44.6)

Which biomarkers do you consider would be necessary to be introduced in your practice?	
Neuron-Specific Enolase	78 (46.4)
Interleukin - 6	70 (41.7)
Procalcitonin	58 (34.5)
Presepsin	43 (25.6)
S100B	42 (25)
Brain natriuretic peptide (BNP)	38 (22.6)
Resistin	35 (20.8)
C - reactive protein	33 (19.6)
D-dimer	33 (19.6)
Troponin	32 (19)
None of the above	14 (8.3)

As reasons for not having large accessibility to bio-markers, the following were mentioned: high costs of biomarker kits (55.4%), limited availability (testing kits are not always available) (41.7%), and concerns over false-positive results (37.5%) ( Table 4).

**Table 4.** Reasons for not using biomarkers currently in clinical practice

**Number of answers = 168**	**n (%)**
What are the biggest challenges you face in using biomarkers in your practice?	
High cost of biomarker kits	93 (55.4)
Limited availability (testing kits are not always available)	70 (41.7)
False positive results	63 (37.5)
The lack of specific guidelines	49 (29.2)
Difficult interpretation of results	9 (5.4)
Lengthy processing times	7 (4.2)

For procalcitonin, PoCT was preferred over the central laboratory, with a median score of 4 (p = 0.001), and the combined use of both methods rated highest at 5. Similarly, presepsin showed a preference for PoCT (median 4) over the central laboratory (median 2, p = 0.001).

## Discussion

This study aimed to assess the use of biomarkers in emergency settings among physicians, highlighting both the benefits and challenges of their application. Most responders worked in county hospitals, emergency departments, and teaching hospitals, aligning with the focus on emergency and academic healthcare environments, reflecting similar trends in current research [16,17,18].

The results of our study underline the frequent daily use, whenewer available, of biomarkers in emergency settings, with PoC testing being the preferred method for most biomarkers when both options were available. This preference is likely driven by the need for more timely and informed decisions [3–4,19]. Biomarkers were particularly valued for their utility in diagnosing sepsis, septic shock, and major cardiovascular conditions, such as acute myocardial infarction and pulmonary embolism. The biomarkers most commonly used in these settings were procalcitonin, troponin, D-dimer, and brain natriuretic peptide (BNP). In terms of the most commonly used biomakers in ED setting, our findings are similar to current literature, which supports the efficacy of these biomarkers in aiding the rapid identification and management of critical conditions in emergency care [2, 8,9,10,11,12, 20].

Physicians identified rapid diagnosis, differential diagnosis, and improved quality of care as the primary benefits of biomarker use, with 86.3% of respondents highlighting these benefits. However, challenges such as high costs, limited availability, and concerns over false-positive results were mentioned, as practical barriers to the widespread use of biomarkers in emergency departments. These obstacles are consistent with existing research that highlights the need for cost-effective and widely available diagnostic tools in emergency settings [19,20,21,22].

Interestingly, the study found significant preferences for specific biomarkers and testing methods. Procalcitonin and presepsin, used in infection-related diagnoses, were preferred for POC testing over central laboratory methods, as probably expected. The preference for POC testing reflects the need for rapid diagnostic results in conditions such as sepsis, where early intervention can significantly improve patient outcomes [12].

Biomarkers such as troponin and D-dimer were highly ranked regardless of the testing method, underscoring their established roles in cardiovascular emergency diagnostics [20]. Moreover, physicians expressed interest in adopting newer biomarkers such as NSE and IL-6, which are useful in evaluation and prognosis of neuronal injury and inflamatory states, respectively [23].

In comparison to previous studies showing that women represent approximately 27% of academic emergency medicine physicians in the U.S. [24–25], our study showed a higher representation of female physicians, with women representing 67.3% of the participants. This notable gender disparity may reflect a broader trend toward increasing female representation in emergency medicine or differences in regional workforce demographics in Romania.

As opposed to the findings of Bennett et al. [25], where the mean age of clinically active emergency physicians was reported to be 50 years with 72% being men, our study registered a markedly younger and more gender-balanced workforce. Specifically, 53.6% of respondents in our survey had between 1–10 years of professional experience, indicating an active engagement of early-career physicians in emergency medical care, highlighting the potential for growth and development within this field. This younger and more diverse group may signify shifting trends in the profession, with newer generations of physicians contributing to a more balanced gender representation in emergency medicine.

Our study has several limitations. First, the sample may not be fully representative for all physicians working in EDs, limiting the generalizability of the findings. Additionally, the study was conducted solely in Romania, which restricts the applicability of the results to other healthcare systems with differing structures and resources. Despite this, our findings are consistent with current literature [20], providing a solid foundation for the extension of this survey to other countries. Future studies could benefit from a more diverse and larger sample size, including multiple countries to enhance the robustness and applicability of the conclusions.

In conclusion to our study, the most used biomarkers by physicians in emergency departments were troponin, D-dimer, BNP/NT-proBNP, and procalcitonin. As for future integration, NSE, IL-6, and procalcitonin were the most ranked ones. However, the main challenges hindering broader implementation include the high costs of biomarker kits, limited availability, and concerns over false-positive results. Further large-scale, multicentric studies are needed to validate the utility of these biomarkers in routine emergency care and to address the challenges.

## Appendix A: Questionnaire Translated into English

### Section 1 – The use of biomarkers in daily practice

1. How frequently do you use biomarkers in your daily practice?
2. How often do you consider it necessary to use biomarkers other than those available in your hospital?
3. The use of biomarkers assists you in:
  - Enhancing the quality of medical care
  - Streamlining patient flow
  - Avoiding the administration of unnecessary treatments
  - Rapid diagnosis of a condition
  - Prognosis
  - Assessing the severity of a condition
  - Differential diagnosis
4. What is the method for biomarker testing in the department where you work?
  - The hospital’s central laboratory
  - The department’s own laboratory with Point of Care (POC) devices
  - Both the hospital’s central laboratory and the department’s own POC laboratory
5. How frequently do you use Point of Care biomarkers?
6. How often do you use biomarkers for the diagnosis of the following critical conditions?
  Sepsis and septic shock
  Acute myocardial infarction
  Pulmonary embolism
  Aortic dissection
  Acute heart failure
  Traumatic brain injury
  Stroke
  Cerebral hypoxia
7. Please rank the following biomarkers according to their utility in your daily practice:
  C-reactive protein
  Procalcitonin
  Presepsin
  Troponin
  D-dimer
  Brain natriuretic peptide (BNP) or N-terminal pro b-type natriuretic peptide (NT-proBNP)
  Interleukin-6
8. Which biomarkers do you consider would be useful to introduce in your hospital?
  Procalcitonin
  Troponin
  D-dimer
  C-reactive protein
  Presepsin
  NT-proBNP
  Interleukin-6
  Neuron-Specific Enolase
  S100B
  Resistin
9. What are the biggest challenges you face in using biomarkers in your practice?
  - False positive results
  - High cost of biomarker kits
  - Limited availability (testing kits are not always available)
  - Lengthy processing times
  - Difficult interpretation of results
  - Lack of specific guidelines

### Section 2 – Demographic and Professional Data

10. Professional category:
  - Senior physician
  - Junior physician
  - 5^th^ year resident
  - 4^th^ year resident
11. Specialty:
  - Emergency Medicine
  - Family Medicine
  - Anaesthesia and Intensive Care
  - Paediatrics
  - Other specialty
12. Professional experience (including residency):
  - 1–5 years
  - 6–10 years
  - 11–15 years
  - 16–20 years
  - 21 years or more
13. Gender:
  - Male
  - Female
  - Prefer not to disclose
14. Age:
  - 20–30 years
  - 31–40 years
  - 41–50 years
  - 51–60 years
  - 61–70 years
  - Over 71 years
15. Type of emergency service where you work:
  - Emergency Department (UPU)
  - Emergency Reception Unit (CPU)
16. Type of work setting:
  - Public system
  - Private system
  - Both
17. Type of hospital where you work:
  - University
  - County
  - Municipal
  - City
  - Military Hospital
  - Private Hospital
  - Other hospital
18. Is the hospital where you work a university hospital (involved in training residents/students)?
  Yes
  No
